# Onion and Apple Functional Ingredients Intake Improves Antioxidant and Inflammatory Status and Vascular Injury in Obese Zucker Rats

**DOI:** 10.3390/antiox11101953

**Published:** 2022-09-29

**Authors:** Claudia Balderas, Javier Angulo, Alejandro Sevilleja-Ortiz, Concepción Peiró, Susana Vallejo, Pilar Dongil, Begoña de Ancos, Concepción Sánchez-Moreno

**Affiliations:** 1 Institute of Food Science, Technology and Nutrition (ICTAN), Spanish National Research Council (CSIC), ES-28040 Madrid, Spain; 2 Ramón y Cajal Institute for Health Research (IRYCIS), ES-28034 Madrid, Spain; 3 Pharmacology Department, Faculty of Medicine, Universidad Autónoma de Madrid (UAM), ES-28029 Madrid, Spain

**Keywords:** functional foods and ingredients, lipid profile, antioxidant enzymes, oxidative stress, vascular injury biomarkers, metabolic hormones, endothelial function, NLRP3, NFKβ1 and COX2 gene expression

## Abstract

The objective of this study was to investigate the effects of onion and apple functional ingredients in homozygous (fa/fa) obese Zucker rats. Rodents were fed three diets: standard diet [obese control (OC) group], standard diet containing 10% onion [obese onion 10% (OO) group] and standard diet containing 10% apple [obese apple 10% (OA) group] for 8 weeks. Food intake and body weight gain were higher in obese than in lean rats. Food efficiency was lower in OO and AO groups compared with OC group. Within the obese groups, total cholesterol, LDL-cholesterol, triacylglycerols, glucose, insulin and triglyceride-glucose index were lower in OO group than in OC group, and HDL-cholesterol was higher in OO group than in OC group. In general, antioxidant activity (ABTS^•+^ and FRAP), antioxidant enzyme activities (CAT, SOD, GPx), GSH/GSSG ratio, nitrate/nitrite and GLP-1 increased in OO and OA groups compared with OC. Oxidative stress biomarkers, namely protein carbonyls, 8-hydroxy-2′-deoxyguanosine, 8-epi-prostaglandin F_2α_, inflammatory and vascular injury biomarkers (PAI-1, TIMP-1, VEGF, sICAM-1, sE-Selectin, MCP-1) and leptin, were lower in OO and OA groups than in OC group. Endothelial impairment was partially reversed, and superoxide content and gene expression of NLRP3, NFKβ1 and COX2 decreased, in OO and OA groups with respect to OC group. The study demonstrates that high pressure-processed onion and apple functional ingredients administration to obese Zucker rats causes beneficial effects on metabolic health, in particular through improving food efficiency ratio; exerting pronounced lipid-lowering effects; reducing glycemia, insulinemia, and biomarkers of hepatic injury (ALT, AST); improving antioxidant, oxidative stress, inflammatory and vascular injury biomarkers, metabolic hormones, and endothelial function; and decreasing proinflammatory gene expression of NLRP3, NFKβ1 and COX2.

## 1. Introduction

Overweight and obesity are major risk factors for many chronic diseases, including diabetes, cardiovascular diseases and cancer. At least 2.8 million people each year die as a result of being overweight or obese. Eating a healthy diet can help prevent obesity. Among the WHO recommendations to maintain a healthy weight is increasing the consumption of fruit, vegetables, pulses, whole grains and nuts [1]. In this context, the literature and epidemiological data and human intervention studies support that high intake of natural functional foods, such as specific fruits and vegetables which are rich in bioactive compounds (including phenolic compounds), has potential beneficial health effects and is an optimal dietary approach to prevent or manage chronic diseases, such as cardiovascular and cardiometabolic diseases, neurodegeneration, cancer, metabolic syndrome, diabetes type II and obesity [2,3,4,5,6]. Dietary interventions are strongly focused on introducing different functional foods into our dietary pattern. Consumer awareness on health and the risks associated with synthetic ingredients, has led to a great demand for products with added or inherent bioactive compounds. Scientists and the research community have already started focusing on the search/development for new innovative compounds/functional ingredients connected to health promotion and disease risk reduction [7,8]. Phytochemicals derived from plant sources such as fruits and vegetables, nuts and seeds, and spices and herbs, among others, are the major types of functional food ingredients projected to be the fastest-growing segment from 2021 to 2026 [9]. In this sense, innovative food companies are using emerging non-thermal technologies more widely; high pressure treatment stands out as a versatile “cold” processing—suitable for functional ingredient extraction and food processing [10,11]. In addition, freeze-drying is the best dehydration method to preserve nutritional qualities [12,13]. Therefore, the combination of innovative food processing technologies with freeze-drying allows to obtain functional food ingredients of plant derived food products [14]. Onions and apples are an essential source of nutrients and bioactive compounds (such as phenolic compounds) and dietary fibre. Daily onion and apple consumption has long been associated with health-promoting properties, including antimicrobial, antibiotic, antioxidant, analgesic, antiplatelet, antithrombotic, antiinflammatory, antidiabetic, anticarcinogenic, hypolipidemic, antihypertensive, hepatoprotective and immunoprotective effects [15,16,17,18]. There is supporting evidence from in vitro and in vivo studies regarding the potential use of onion and apple bioactive compounds or extracts (leaves, bark, skin or processed products) as effective food ingredients with specific health-beneficial effect beyond their nutritional properties [16,18,19,20,21,22,23]. However, specific effects of functional ingredients from high pressure-processed onion and apple in studies with humans or animal models are less studied. Recently, we showed an improvement in the antioxidant and antiinflammatory response as well as in cardiovascular risk biomarkers and vascular dysfunction promoted by a high pressure-processed onion ingredient in hypercholesterolemic male Wistar rats [24]. Some studies have shown the cholesterol-lowering effect of apple products in animal models [25]. Recently, Yuste et al. (2021) [26] showed cardiometabolic protective effects of both red-fleshed and white-fleshed apples supplementation in hypercholesterolemic rats. In this sense, oxidative stress and inflammation are key processes in the impairment of vascular function related to metabolic diseases [27,28] and represent main factors in the development of cardiovascular diseases in patients with metabolic alterations [29,30]. Thus, based on our previous studies and literature findings, we hypothesized that onion and apple functional ingredients (high pressure-processed) intake will have an impact on plasma lipid levels, antioxidant, inflammatory and vascular injury biomarkers, metabolic hormones, endothelial dysfunction, NADPH oxidase (NOX) activity and NLRP3, NFKβ1 and COX2 gene expression in homozygous (fa/fa) obese Zucker rats. The obese Zucker rat is an established monogenic model of human early-onset, hyperplastic–hypertrophic obesity, that exhibits hyperphagia, hyperinsulinemia, and hyperlipidemia [31] and can provide evidence of the possible metabolic consequences of onion and apple functional food ingredients consumption.

## 2. Materials and Methods

### 2.1. Onion and Apple Powder Preparation

Raw onions (*Allium cepa* L. var. *cepa*, ‘Recas’) and apples (*Mallus pumila* Mill., ‘Golden delicious’) were purchased from a local supermarket (Madrid, Spain). The onions were hand-peeled and cut into 10 mm pieces. The apples were washed, divided into quarters without core, cut into 2 cm pieces with skin and quickly packed. Both cut onion and apple (approx. 120 g) were packaged in very low gas permeability bags (BB3255, Cryovac, Barcelona, Spain) and treated by high pressure (400 MPa/25 °C/5 min and 400 MPa/35 °C/5 min, respectively) (High Pressure Iso-Lab System, model FPG7100:9/2C, Stansted Fluid Power Ltd., Essex, UK). After the high pressure treatment, the onion and apple were frozen with liquid nitrogen, freeze-dried in a lyophilizer (model Lyoalfa, Telstar, S.A., Barcelona, Spain) and pulverized with an ultra-centrifugal mill ZM 200 (Retsch GmbH, Haan, Germany), obtaining a fine powder (final size particle ≤250 μm), and stored at −20 ± 0.5 °C until use. Table 1 shows nutritional composition, phytochemical compounds and antioxidant activity of the onion and the apple powder. Analyses were carried out using the methods described by González-Peña et al. (2013) [32], Colina-Coca et al. (2013) [33] and Colina-Coca et al. (2014) [34].

### 2.2. Animals and Experimental Design

The present study was approved by the Spanish Ministry of Economy, Industry and Competitiveness Advisory Committee (project AGL2016-76817-R) and by the Ethics Committee of the Complutense University of Madrid (Spain) (Reference PROEX 133/16). All experiments were performed in compliance with Directive 2010/63/UE regarding the protection of animals used for scientific purposes. All necessary steps were taken to prevent any potential animal suffering. Twenty-four male, homozygous (fa/fa), obese Zucker rats (Crl:ZUC(Orl)-*Lepr*^fa^) with a body weight of approximately 225 g at the outset, and eight male, heterozygous (fa/+), lean Zucker rats with a body weight of approximately 200 g at the outset, all 7 wk of age, were acquired from Charles River Laboratories, Barcelona, Spain. The animals were housed in groups of four under controlled conditions (12 h light−12 h dark cycle, 22.5 ± 0.5 °C ambient temperature, 50–60% relative humidity). The rats were fed commercial rat pellets (Panlab, SLU, Barcelona, Spain) for 5 days for the adaptation to environmental conditions. The obese Zucker rats were randomly divided into three groups of eight rats each: obese control (OC) group, obese Zucker rats fed a standard diet; obese onion 10% (OO) group, obese Zucker rats fed a standard diet containing 10% onion; and obese apple 10% (OA) group, obese Zucker rats fed a standard diet containing 10% apple. Lean Zucker rats served as a lean control (LC) group: lean Zucker rats fed a standard diet. Three semisynthetic diets were prepared based on the AIN-93M semi-purified rodent diet [35], with comparable levels of gross energy and crude nutrients (Table 2). In OO and OA diets, maize starch and cellulose powder were adjusted to compensate for the addition of onion and apple powder, respectively. The doses for onion and apple powder were selected based on the body surface area normalization method [36] and previous studies [24,37,38], representing an amount of onion and apple that could reasonably be expected to be achieved in the human population having healthy food habits. Water and food were provided ad libitum over the 8-week experimental feeding trial. Body weight and faecal weight were recorded weekly and food intake was recorded daily. The food efficiency ratio, i.e., the relationship between body weight gain and food intake, was calculated using the formula: 100 × [body weight gain (g)/total food intake (g dry matter)]. The apparent diet digestibility, i.e., the percentage of food digested and absorbed, was calculated using the formula: 100 × [food intake (g dry matter)–faecal weight (g dry matter)/food intake (g dry matter)]. Both parameters are frequently employed in animal nutrition studies to evaluate food digestibility and utilisation.

### 2.3. Urine, Faeces, Blood and Organs Sampling

Once a week, animals were housed individually to collect urine and faeces. At the end of the feeding trial, in order to avoid inter-assay variations that could affect the comparison of data from the different groups, animals in fasting conditions were euthanized by decapitation, taking randomly one animal at a time, of each one of four groups. Trunk blood was collected into tubes with EDTA as anticoagulant. Plasma was recovered after centrifugation (1500× *g*, 15 min) at 4 °C and immediately stored at −80 °C until analysis. The whole organs and tissues (brain, cerebellum, liver, spleen, lungs, pancreas, kidneys, heart, testis, stomach, small intestine, large intestine, muscle, white and brown adipose tissue) were collected and weighed before being frozen in liquid nitrogen and stored at −80 °C until analysis.

### 2.4. Plasma Lipid Concentrations and Biochemical Parameters

Total cholesterol (TC), HDL-cholesterol (HDL-C), LDL-cholesterol (LDL-C), triacylglycerols (TG), glucose, urea, uric acid, creatinine, albumin, alanine aminotransferase (ALT/GPT, EC 2.6.1.2), aspartate aminotransferase (AST/GOT, EC 2.6.1.1), gamma-glutamyl transpeptidase (GGT, EC 2.3.2.2) and total bilirubin were measured in rat plasma samples using a Cobas Mira Plus analyzer (Roche Diagnostics Ltd., Rotkreuz, Switzerland). Insulin was measured using an ELISA kit (Cat. No. 10-1250-01, Rat Insulin ELISA, Mercodia AB, Uppsala, Sweden). Atherogenic indexes (AI) were calculated as follows: AI (1) = LDL-C/HDL-C, AI (2) = TC/HDL-C. The following indexes were also calculated: HOMA-IR = [Fasting Insulin (microU/mL) × Fasting Glucose (mmol/L)]/22.5, Triglyceride–Glucose Index = Ln[Fasting Plasma Triacylglycerols (mg/dL) × Fasting Glucose (mg/dL)/2] and Glucose/Insulin Ratio = Fasting Glucose (mg/dL)/Fasting Insulin (microU/mL).

### 2.5. Plasma Antioxidant Activity

2,2′-azinobis(3-ethylbenzothiazoline-6-sulfonic acid) radical cation (ABTS^•^^+^) scavenging capacity and ferric reducing antioxidant power (FRAP) in plasma were measured by the methods described in González-Peña et al. (2013) [32], slightly modified. ABTS^•^^+^ and FRAP values were expressed as μmol Trolox Equivalents (TE)/L.

### 2.6. Antioxidant Enzyme Activities

Evaluation of superoxide dismutase (SOD, EC 1.15.1.1), catalase (CAT, EC 1.11.1.6) and glutathione peroxidase (GPx, EC 1.11.1.9) activities in erythrocytes and liver were measured using the Superoxide Dismutase Assay Kit (Cat. No. 706002), Catalase Assay Kit (Cat. No. 707002) and Glutathione Assay Kit (Cat. No. 703102) (Cayman Chemical Company, Ann Arbor, MI, USA), respectively, following the vendor’s instructions. The CAT and GPx activities were expressed in nmol/min/mL in erythrocytes and nmol/min/mg protein in liver.

The SOD activity was expressed in U/mL in erythrocytes and U/mg protein in liver.

The total protein content in the liver homogenates was measured using a commercial protein assay kit (Bio-Rad Protein Assay Kit, Bio-Rad Laboratories, Alcobendas, Madrid, Spain).

### 2.7. Oxidative Stress Biomarkers

Erythrocyte and liver glutathione disulphide and glutathione were measured using the Glutathione Assay Kit (Cat. No. 703002). The glutathione disulphide and glutathione contents were expressed as µmol/mL in erythrocytes and µmol/mL/mg in liver. Protein carbonyls were measured in plasma and liver homogenates according to the manufacturer’s instructions (Cat. No. 10005020, Cayman Protein Carbonyl Colorimetric Assay Kit). Urine 8-hydroxy-2′-deoxyguanosine, urine 8-*epi*-prostaglandin F_2α_ and plasma and urine nitrate/nitrite concentrations were determined using DNA/RNA Oxidative Damage (Clone 7E6.9) ELISA Kit (Cat. No. 501130), 8-isoprostane ELISA Kit (Cat. No. 516351) and Nitrate/Nitrite Colorimetric Assay Kit (Cat. No. 760871), respectively (Cayman Chemical Company, Ann Arbor, MI, USA).

### 2.8. Plasma Vascular Injury Biomarkers and Metabolic Hormone Concentration

Milliplex^®^ Map Rat Vascular Injury Magnetic Bead Panel 1 (RV1MAG-26K) selecting the plasminogen activator inhibitor 1 (PAI-1), tissue inhibitor of matrix metalloproteinases type 1 (TIMP-1) and vascular endothelial growth factor (VEGF), and Milliplex^®^ Map Rat Vascular Injury Magnetic Bead Panel 2 (RV2MAG-26K) combining the adhesion molecules soluble intercellular adhesion molecule-1 (sICAM-1) and soluble E-selectin (sE-selectin), and the adipocytokine adiponectin, were adequately used for the quantification of vascular injury biomarkers in rat plasma (EMD Millipore Corporation, Billerica, MA, USA). Milliplex^®^ Map Rat Metabolic Magnetic Bead Panel Kit (RMHMAG-84K) was used for the quantification of monocyte chemoattractant protein-1 (MCP-1), glucagon-like peptide-1 (GLP-1) and leptin. Plasma levels were measured using Luminex xMAP^®^ technology (MAGPIX^®^ System) according to the manufacturer’s recommendations.

### 2.9. Vascular Reactivity in Mesenteric Arteries

Endothelium-dependent relaxation induced by acetylcholine (ACh) as well as endothelium-independent relaxation induced by sodium nitroprusside (SNP) were evaluated in rat mesenteric arteries (RMA). Third branch mesenteric arteries were dissected by carefully removing the adhering fat tissue. Arterial ring segments (1.7–2.0 mm long) were subsequently mounted on microvascular wire myographs (J.P. Trading, Aarhus, Denmark) for isometric tension recordings as previously described [39]. The vessels were allowed to equilibrate for 30 min in Krebs–Henseleit solution (KHS) continuously bubbled with 95% O_2_/5% CO_2_ mixture to maintain a pH of 7.4. Passive tension and internal circumference of vascular segments, when relaxed in situ under a transmural pressure of 100 mmHg (L100), were determined. The arteries were then set to an internal circumference equivalent to 90% of L_100_, at which the force development was close to maximal. To assess vessel viability, preparations were then exposed to 125 mM K^+^ (KKHS, equimolar substitution of NaCl for KCl in KHS) and the contractile response was measured. After a washout and stabilization period, rat arteries were contracted with 1–3 μM norepinephrine (NE, 80% of KKHS-induced contraction, approximately) and relaxation responses were evaluated by cumulative additions of ACh (1 nM to 30 μM) to the chambers.

Experiments were run in parallel. For determining the effects of superoxide scavenging and inhibition of NOX on endothelium-dependent responses, arterial segments were incubated for 30 min with TEMPOL (10 μM) or VAS-2870 (10 μM), respectively. Concentration-response curves to ACh in arterial segments from the same animal that previously received only vehicle (distilled water) were considered as controls for the evaluation of the effects of these treatments. For the evaluation of the impact of onion and apple lyophilized powder intake on endothelium-independent vasodilatations, cumulative additions of SNP (1 nM to 100 μM) were added on NE-precontracted arterial segments from lean control (LC) group and obese (OC, OO, OA) groups.

### 2.10. Detection of Superoxide Anion Generation

Rat mesenteric arteries specimens and heart left ventricles were removed and dissected free from adherent connective tissue and fat. Tissues were then immersed in saccharose (30% *w/v*), embedded in OCT and stored at −80 °C until immunofluorescence assay. OCT blocks were cut in a cryostat and mounted on polylysine-coated glass slides. In situ superoxide anion production was measured using the fluorescent dye dihydroethidium (DHE) as described previously [40]. Briefly, 6 μm-thick sections of rat mesenteric arteries and hearts were incubated with DHE (4 μM; Invitrogen, Life Technologies Corporation, Eugene, OR, USA) for 30 min at 37 °C in a humidified chamber protected from light. In the presence of superoxide anion, DHE is oxidized to ethidium that yields bright red fluorescence. After washing with PBS plus 0.05% Triton X-100, sections were mounted and visualized by fluorescence microscopy (Olympus BX51, Olympus Corporation, Tokyo, Japan). The ratio of nuclei showing positive red signal with respect to total nuclei (counterstained with 300 nM diamidino-2-phenylindole (DAPI), Life Technologies) was determined with Image J imaging software (McBiophotonics Image J, NIH, Bethesda, MD, USA).

### 2.11. RNA Isolation and Quantitative Real Time PCR (RT-PCR) Assay

Relative gene expression of NLRP3, NFKβ1 and COX2 were measured in aortic tissues, as previously described [41]. Total aorta RNA was extracted by using NZYol (NZYTech, Lisboa, Portugal). RNA concentration and purity were analysed spectrophotometrically using NanoDrop 2000 (Thermo Fisher Scientific, Waltham, MA, USA). Reverse transcription (RT) was carried out using the kit First-Strand cDNA Synthesis (NZYTech, Lisboa, Portugal). 500 ng of total RNA was reverse transcribed to cDNA following the manufacturer’s instructions. The mRNA expression levels were measured using qRT-PCR based SYBR Green technology (Bio-Rad) developed by the 7500 Fast Real-Time PCR System (Thermo Fisher Scientific, Waltham, MA, USA). For all analyses, qRT-PCR was carried out with iTaq Universal SYBR Green Supermix (Bio-Rad) and the specific oligonucleotides for the genes *Nlrp3*, *Nfkβ1* and *Cox2* (Sigma-Aldrich, St. Louis, MO, USA). Amplification conditions included an initial denaturation step at 95 °C for 20 s followed by 40 cycles at 95 °C for 3 s and 60 °C for 30 s. The quantification of the relative gene expression was determined by the 2^−ΔΔCt^ method and the mRNA levels were normalized to the reference gen *ARNr 18S*. Each sample was amplified in triplicate for each gene. Data were analysed with the ABI PRISM 1.7 analysis software (Applied Biosystems, Waltham, MA, USA).

### 2.12. Statistical Analysis

Results are reported as mean values with their standard deviation (SD). Data were analysed using one-way ANOVA. In order to verify the homogeneity of the variances a Levene’s test was applied. Tamhane’s T2 (equal variances not assumed) or Bonferroni (equal variances assumed) post hoc tests were used to determine differences between groups (*p* < 0.05). Mean values within the same group (LC, OC, OO, OA) at the start (baseline or week 0) and the end of the experimental trial (week 8) for some parameters (urine 8-hydroxy-2′-deoxyguanosine, urine 8-*epi*-Prostaglandin F_2α_, urine nitrate/nitrite) were statistically tested using Student’s *t* test (*p* < 0.05). Correlations were carried out using Pearson’s *r* correlation. Analyses were performed using the IBM SPSS Statistics 27 (SPSS Inc., an IBM Company, Armonk, NY, USA). For vascular reactivity and detection of superoxide anion generation, data are shown as mean ± SEM. Relaxation responses are expressed as the percentage of maximum relaxation induced by the addition of papaverine (100 μM) at the end of the experiment. pEC_50_ is defined as the -log M of the concentration required to obtain 50% of maximal relaxation. To compare complete concentration-response curves, a two-factors analysis of variance (ANOVA) test was applied using StatView software for Apple computers (SAS, Cary, NC, USA). This statistical test compares concentration-response curves, including all concentrations in the analysis. When more than two concentration-response curves were compared, Bonferroni correction was applied. Other data were compared by one-factor ANOVA followed by Student–Newmann–Keuls test for multiple comparisons. For RT-PCR, data are expressed as mean ± SEM of five independent experiments; * *p* < 0.05 vs. LC; # *p* < 0.05 vs. OC by one-way ANOVA followed by Sidak post-hoc test.

## 3. Results and Discussion

### 3.1. Weight Gain and Feed Intake

Initial and final body weights, body weight gain, food intake and faecal weight were lower in group LC than in groups OC, OO and OA (*p* < 0.05, Table 3). Within the obese groups, food efficiency ratio was higher in group OC. Apparent diet digestibility did not differ between the four groups (*p* < 0.05, Table 3) and food efficiency ratio was significantly lower (decrease by 22%) in OO and OA groups compared with OC group, which indicate a positive effect of dietary treatments. These findings in the reduction of the food efficiency ratio in rats fed the onion and apple lyophilized powder are consistent with previous studies showing that administration of cinnamon extract powder in hypercholesterolemic adult male rats [42], Stachys sieboldii root powder in Sprague Dawley rats feed a high-fat and high-cholesterol diet [43], and white kidney bean flour in male albino rats [44] decreased the food efficiency ratio.

### 3.2. Plasma Lipid Concentrations and Biochemical Parameters

Plasma concentrations of total cholesterol, LDL-cholesterol, triacylglycerols, glucose, insulin and triglyceride–glucose index were lower in group LC than in groups OC, OO and OA (*p* < 0.05, Table 4). Within the obese groups, total cholesterol, LDL-cholesterol, triacylglycerols, glucose, insulin and triglyceride–glucose index were lower in OO group than in OC group, and HDL-cholesterol was higher in OO group than in OC group. Consequently, AI (1) was lower in OO group than in OC group, and AI (2) and HOMA-IR were lower in OO and OA groups than in OC group (*p* < 0.05, Table 4). Plasma concentrations of ALT, AST and GGT were higher in OC group than in LC, OO and OA groups. These variables did not differ between OO and OA groups. Plasma concentrations of albumin and total bilirubin were lower in group LC than in groups OC, OO and OA. Glucose/insulin ratio and plasma concentration of urea, uric acid, creatinine and albumin did not differ between the four groups (*p* < 0.05, Table 4).

The onion diet showed an unequivocal and significant plasma cholesterol-lowering (−35% in total cholesterol, −14% in LDL-cholesterol) and triacylglycerol-lowering (−22%) effects in obese Zucker rats, whereas the apple diet showed lower effects (−25% in total cholesterol, −5% in LDL-cholesterol, −9% in triacylglycerols). Although statistical significance was not reached, a clear tendency towards decreased values was found in OA rats. The LDL-cholesterol/HDL-cholesterol ratio (AI (1)) was particularly high in obese Zucker rats fed the standard diet (0.41) compared with lean Zucker rats (0.19), and it was strongly depressed in rats fed the onion diet to 0.261 (−37%); however, in obese rats fed the apple diet, the AI (1) decrease was less pronounced (−16%). These differences could be attributed to the different phytochemical composition between onion and apple lyophilized powder. Onion powder was rich in quercetin and isorhamnetin glucosides and sulphides compounds, whereas these compounds were not detected in apple powder. Supporting the present finding, sulphur compounds, such as cycloalliin, *S*-methyl-L-cysteine, *S*-propyl-L-cysteine sulphoxide, dimethyl trisulphide, and especially *S*-methyl-L-cysteine sulphoxide, were reported to be effective in inhibiting formation of oil drop in the cells, suggesting that these compounds may be involved in the antiobesity effect of the onion extract [45]. Therefore, the present results are in agreement with those reported in the literature showing lipid-lowering effects of different *Allium cepa* and apple products or extracts [15,46,47]. Regarding glycemia and insulinemia, a marked and significant reduction in plasma glucose and insulin in OO group (−35% and −49%, respectively) compared to OC group was found; meanwhile, plasma glucose and insulin levels in OA group were lower than in OC group, but less pronounced (−23%, −34%; respectively). Reductions in plasma glucose concentrations may be particularly relevant given the relationship between cardiovascular events and all-cause mortality predicted by postprandial blood glucose [48]. These results are in agreement with those reported by Cheng et al. (2020) [49] evaluating the metabolic effects of green tea polyphenols (GTP) in high-fat diet fed Zucker fatty rats. In this study, significantly reduced fasting serum glucose, insulin levels and HOMA-IR were found in GTP-treated animals compared with Zucker fatty rats control group. In addition, Yoshinari et al. (2012) [45] found that feeding Zucker diabetic fatty rats with onion extract improved fasting blood glucose and HOMA-IR. On the other hand, plasma levels of ALT, AST and GGT were significantly different for OC and LC rats. The levels of enzymes and proteins in the blood that are produced can be used to analyse liver health. In this sense, in the current study, the levels of the three liver enzymes were significantly decreased in OO and OA groups (−47%, −27% and −22%, respectively, in OO group; and −39%, −25% and −15%, respectively, in OA group) compared with OC group. High plasma levels of liver enzymes ALT, AST and GGT increase the risk of disease and all-cause mortality and reflect liver injury. This finding is consistent with a recent study in which Colina-Coca et al. (2017) [24] reported a decrease in circulating levels of ALT and AST enzymes in hypercholesterolemic Wistar rats fed with an onion ingredient compared with high-cholesterol fed rats. This is a key finding of this study; onion and apple ingredients protected liver damage and genetic obesity, evidenced by improved plasma levels of hepatic enzymes. These results indicate that onion and apple supplementation could be helpful in the prevention/treatment of hepatic steatosis in obese Zucker rats. In addition, different studies have reported that the intake of vegetable-enriched diets was associated with lowering effects in liver enzymes in rodent models [49,50,51].

### 3.3. Antioxidant, Inflammatory and Vascular Injury Biomarkers and Metabolic Hormones

Plasma antioxidant activity was lower in OC group than in LC, OO and OA groups without significant differences between these three groups. Plasma ABTS^•+^ and FRAP values increased by 14% and 47%, respectively, in OO and OA groups compared with OC group (Table 5). Two methods were used to measure the plasma overall antioxidant activity. The FRAP assay is based on the conversion of ferric to ferrous form by antioxidants present in the plasma and does not measure the thiol group containing antioxidants. The ABTS assay measures the relative ability of antioxidants to scavenge the ABTS^•+^ generated in aqueous phase and is reactive towards most antioxidants including thiols. In line with the present results, Codoñer-Franch et al. (2013) [52] showed that supplementation with an apple snack in tamoxifen-treated rats significantly increased the plasma antiradical activity measured by ABTS^•+^ and FRAP methods. On the contrary, Aprikian et al. (2002) [15] reported that the apple diet did not affect the plasma FRAP value in lean or obese rats. Del Pino-García et al. (2016) [53] studied the bioavailability of phenolics contained in a powdered red wine pomace seasoning (RWPS) and its beneficial effects after acute and short-term supplementation in healthy Wistar rats. They found plasma FRAP and ABTS values significantly higher at 4 h post RWPS intake compared to samples collected before the intervention; however, after daily RWPS consumption for 4 weeks no significant differences between control and supplemented rats were found. In contrast, the RWPS supplementation in streptozotocin-induced diabetic rats for 4 weeks increased plasma FRAP and ABTS values compared to the non-supplemented rats [54], and in spontaneously hypertensive rats (SHR) it increased plasma FRAP compared to the non-supplemented rats [55].

Erythrocyte catalase (CAT) activity was higher in OO group (increase by 5%) than in OC and OA groups. Liver CAT activity did not differ within the obese groups (OC, OO, and OA) and was lower than in LC group. Erythrocyte superoxide dismutase (SOD) activity was higher in OO (increase by 37 %) and in OA (increase by 18%) groups than in OC group. Liver SOD activity was lower in obese groups than in LC group. Erythrocyte and liver glutathione peroxidase (GPx) activity were higher in OO (increase by 43% and 32%, respectively) and in OA (increase by 28% and 28%, respectively) groups than in OC group. To prevent the oxidative damage caused by the increase in oxidative stress the organism has built an antioxidant defence system, which includes antioxidants that can remove harmful active metabolites and detoxify antioxidant enzymes, protecting cells and organs from all kinds of oxidative damage induced by free radicals. Intracellular antioxidant enzymes play an important role in cellular antioxidant defences. Major enzymes associated with the quenching of reactive oxygen species (ROS) are CAT, SOD and GPx. CAT, SOD and GPx are prone to oxidative damage and sensitive to inactivation by ROS. In the present study, erythrocyte and liver CAT, SOD and GPx activities were lower in OC group compared with LC group, in accordance with the hyperinsulinemia, hyperlipidemia and obesity-induced oxidative stress that exhibit this genetic obesity model [56,57]. The results found in erythrocyte and liver antioxidant enzymes activities suggest an important role for the onion and apple ingredients in preventing obesity-mediated redox imbalance in obese Zucker rats maintaining or enhancing the activity of enzymatic antioxidants and/or inhibiting the generation of free radicals.

GSH is a sulphur-containing tripeptide formed in the liver and is a water-soluble cellular antioxidant and an enzyme co-factor. It is present in reduced (GSH) and oxidized form (GSSG), the ratio of which is considered a good marker of the redox status within the cell. Erythrocyte glutathione/glutathione disulphide (GSH/GSSH) ratio was lower in OC group than in LC, OO and OA groups. Erythrocyte GSH/GSSH ratio increased by 43% and 68%, respectively, in OO and OA groups compared with OC group. Liver glutathione/glutathione disulphide (GSH/GSSH) ratio was lower in OC group than in LC, OO and OA groups. Liver GSH/GSSH ratio increased by 28% and 26%, respectively, in OO and OA groups compared with OC group. The changes in erythrocyte and liver GSH/GSSH ratio agreed with those reported by Deka et al. (2021) [58] showing a substantial increase in GSH/GSSH ratio in high-fat diet-fed male Sprague Dawley rats supplemented with *Allium hookeri* extract. The results suggest that sulphide compounds may have played an important role in maintaining the intracellular redox potential via eliminating the reactive intermediate, either directly or by stimulating the biosynthesis of the GSH pool.

Plasma and liver protein carbonyls were lower in OO (decrease by 15% and 25%, respectively) and OA (decrease by 28% and 24%, respectively) groups than in OC group. Oxygen radicals have been implicated as an important cause of oxidative modification of proteins, which may lead to their inactivation and rapid degradation. Among the various oxidative modifications of amino acids in proteins, carbonyl formation may be an early marker for protein oxidation [59], and resulting carbonylation of proteins an important hallmark of oxidative stress. In this study, onion and apple supplementation caused a decreased in protein carbonyls compared with OC group. These results are in agreement with those reported by other authors after feeding different animal models with diverse plant-derived diets [24,52,60,61,62].

The oxidation of the DNA bases by ROS can occur either directly in the genomic DNA strands or indirectly in the nucleotide pool, from where the modified bases are introduced into the genomic DNA during replication or repair. Oxidative DNA damage can result from reactions with purine and pyrimidine bases, deoxyribose or phosphodiesters. 8-hydroxy-2′-deoxyguanosine is a notable biomarker used to quantify oxidative DNA damage in animals and humans [63]. Urine 8-hydroxy-2′-deoxyguanosine at baseline (week 0) did not differ between the obese groups. Urine 8-hydroxy-2′-deoxyguanosine at the end of the experimental trial (week 8) was lower in OO and OA groups than in OC group (decrease by 23% and 18%, respectively). In this sense, Azuma et al. (2010) [64] evaluated the tolerable level of dietary quercetin for exerting its antioxidative effect in high-cholesterol-fed rats, using quercetin-containing diets and onion diets, from the viewpoint of a safety assessment. These authors reported that, in the case of onion intake, none of the tested onion diets elevated the urinary 8-oxo-7,8-dihydro-2′-deoxyguanosine levels after intake, and one of the onion diets evaluated significantly lowered it compared with the level before intake. In agreement with those findings, Pradeep and Srinivasan (2018) [65] reported that renal 8-hydroxy-2-deoxyguanosine, its excretion, DNA fragmentation and mitochondrial DNA deletion were significantly annulled in streptozotocin-induced diabetic rats by dietary interventions with fenugreek seeds and onion. In addition, Codoñer-Franch et al. (2013) [52] confirmed their hypothesis that the consumption of an apple snack rich in antioxidants would counteract liver toxicity produced by tamoxifen administration in rats, reducing significantly the liver 8-hydroxy-2′-deoxyguanosine levels.

The F2-isoprostanes are formed by the free radical catalysed peroxidation of phospholipid bound arachidonic acid and released into the circulation. Levels of both urinary and plasma F2-isoprostanes have correlated with coronary artery disease risk factors. They are formed, mostly within cell membranes, out of fatty acids, predominantly arachidonic acid (but also out of eicosapentaenoic and docosahexaenoic acid), are released by the aid of phospholipases and are similar in structure to prostaglandins [66]. In the current study, urine 8-*epi*-prostaglandin F_2α_ at baseline did not differ between the four groups (*p* < 0.05, Table 5). The levels of urine 8-*epi*-prostaglandin F_2α_, which are products of free radical-mediated in vivo oxidation of arachidonic acid different from COX-derived oxidized prostaglandins, such as PGE_2_, were elevated in OC group compared with LC group at week 8. Urine 8-*epi*-prostaglandin F_2α_ at week 8 was lower in OO and OA groups than in OC group (decrease by 57% and 58%, respectively), pointing out a significant effect of the onion and apple powder in the generation of these lipid peroxidation biomarkers. In this regard, higher prevention of lipid peroxidation (urinary F2-isoprostanes) and improved nitric oxide bioavailability were observed in samples collected at 4 h and between 3 and 6 h in the study by Del Pino-García et al. (2016) [53] regarding the RWPS beneficial effects after acute supplementation in healthy Wistar rats. The results suggest an important antioxidant role of the phenolic metabolites generated by the action of colonic microbiota. The RWPS supplementation in streptozotocin-induced diabetic rats [54] and SHR [55] for 4 weeks decreased plasma F2-isoprostanes compared to the non-supplemented rats. Additionally, the level of F2-isoprostanes and GSH/GSSG indicated a reduction in liver oxidative stress by the consumption of strawberry aqueous extract (rich in phenolic content) in male Sprague Dawley rats subjected to lipopolysaccharide-induced liver injury [67].

Plasma nitrate (NO_3_^−^) and nitrite (NO_2_^−^), end products of NO metabolism, were measured as markers of NO status. Plasma nitrate/nitrite was lower in OC group than in LC, OO and OA groups, without significant differences between these three groups. Since NO metabolites reflect the amount of systemic NO, the content of 24-h urinary nitrate/nitrite was measured. Urine nitrate/nitrite at baseline did not differ within the obese groups (OC, OO, and OA) and was lower than in LC group. Urine nitrate/nitrite at week 8 was higher in OO and OA groups than in OC group (increase by 34% and 15%, respectively). This is an interesting finding as nitrate/nitrite are critical mediators to limit oxidative injury and inflammation [68]. The formation of NO from NO_3_^−^ and NO_2_^−^ via a reductive ‘NO_3_^−^–NO_2_^−^–NO’ pathway and resulting in vasodilation is now an established complementary route to traditional NOS-derived vasodilation [69]. In this sense, in the study by Del Pino-García et al. (2017) [55], 50% higher nitrate/nitrite levels were found in SHR supplemented with RWPS than in untreated SHR. Also, Maneesai et al. (2021) [70] reported that *Clitoria ternatea* extract improved plasma nitric oxide metabolites (NOx) in *N*_ω_-Nitro-L-arginine methyl ester hydrochloride (L-NAME)-induced hypertensive rats. In this same animal model, the treatment with genistein restored the reduced plasma NOx concentrations [71]. Mukai and Sato (2009) [72] reported the level of 24-h urinary NOx was significantly higher in the polyphenol-containing azuki bean extract (ABE)-treated SHR than in the untreated SHR, indicating that ABE treatment upregulated NO production. All these results are in agreement with several studies reporting that treatments with flavonoids such as quercetin suppress the development of hypertension associated with a reduced oxidant status due to their antioxidant properties.

Evidence regarding obesity-induced oxidative stress is derived from several clinical studies, which have established correlations of biomarkers, or end-products of free radicals-mediated oxidative stress (lipid peroxidation or protein carbonylation products), with body mass index. In addition, there is a known and confirmed connection between inflammation, oxidative stress and atherosclerosis. Inflammation plays a key role in vascular stiffening and other pathologies that induce vascular damage [73]. In this regard, several biomarkers are being suggested as the link between obesity, insulin resistance, cardiovascular disease and metabolic syndrome. Among them, in the present study, plasma PAI-1, TIMP-1, VEGF, sICAM-1, sE-selectin, MCP-1 and adiponectin were measured as inflammatory and vascular injury biomarkers. All plasma inflammatory and vascular injury biomarkers were lower in LC group than in OC group (*p* < 0.05, Table 5). PAI-1 was lower in OO and OA groups than in OC group (decrease by 17% and 15%, respectively). TIMP-1 was lower in OO and OA groups than in OC group (decrease by 26% and 16%, respectively). VEGF was lower in OO and OA groups than in OC group (decrease by 24% and 17%, respectively). sICAM-1 was lower in OO and OA groups than in OC group (decrease by 18% and 41%, respectively). sE-selectin was lower in OO and OA groups than in OC group (decrease by 13% and 18%, respectively). MCP-1 was lower in OO and OA groups than in OC group (decrease by 25% and 33%, respectively). Adiponectin did not differ within the obese groups (OC, OO, and OA) and was lower (decrease by 14%) than in LC group. The onion and apple diets reduced PAI-1 and TIMP-1 concentration in the plasma. Belobrajdic et al. (2011) [74] found that PAI-1 was elevated in the plasma of obese rats, and a wheat bran diet compared to the control group reduced PAI-1 to levels seen in the lean rats. The authors report that this change in PAI-1 could not be explained by the secretion rates of PAI-1 from subcutaneous or visceral adipose tissue, suggesting that it was likely that an alternative site of PAI-1 synthesis, the liver, was responsible for regulating circulating PAI-1 levels. As found in the present study, association and cohort studies also show an inverse association between high fibre diets and PAI-1 levels, confirming the role of diets high in fibre in the reduction of inflammatory markers [74]. In addition, plasma levels of PAI-1 are more closely related to the degree of liver steatosis than to the fat accumulation in adipose tissue [75]. A strong association between plasma ALT and AST and PAI-1 concentration reinforces this fact in the present study. In this line, Martins et al. (2010) [76] reported that conjugated linolenic acid promoted lower PAI-1 serum concentrations in obese Zucker rats regardless of the fat source, indicating also a concomitant decrease in AST, although not in ALT. TIMP-1 is a crucial regulator of extracellular matrix degradation and circulating TIMPs have been shown to be altered in several cardiovascular diseases [77]. An early study shows that increased TIMP-1 levels are a risk factor for mortality among congestive heart failure patients [78]. Recently, Jiang et al. (2022) [79] found that Siberian onions decreased serum transaminase levels and oxidative stress, and regulated the balance of the extracellular matrix in CCl4-induced mice, including α-smooth muscle actin (α-SMA), type I collagen, and TIMP-1, suggesting that Siberian onions reduced the release of inflammatory factors and regulated apoptosis-associated proteins, which is related to the inhibition of signal transducer and activator of transcription 3 (STAT3) phosphorylation. Additionally, considering that TIMP-1 is an inhibitor of MMP-1 in tissue and the amount of type I collagen is tightly regulated by balance between MMP-1 and TIMP-1, Cho et al. (2010) [80] demonstrated that onion extract and quercetin play a role in the antifibrotic effect and promotion of wound healing in skin through up-regulation of MMP-1 expression. The current knowledge on the regulation and functions of VEGFs, and their role in lipid metabolism and atherosclerosis, points to their high potential as biomarkers for prognosis, monitoring CVDs progression and severity. In line with this, scientific evidence supports their use as antiatherosclerosis therapy based on the studies about drugs and natural compounds that directly or indirectly affect VEGFs’ level of expression or signalling [81]. Recently, it was shown that peonidin and petunidin, two anthocyanins naturally occurring in berries and grapes, regulate angiogenesis and atherogenesis. In particular, in a TNF-α stimulated proinflammatory environment, peonidin and petunidin prevent monocyte adhesion to HUVECs, and reduce VCAM-1, E-selectin and VEGF production [82]. One of the possible mechanisms of regulation of VEGF’s expression and signalling could rely on the direct hydrophobic interaction between VEGF and polyphenol molecules, causing a change in the secondary structure of the protein, thus inhibiting further VEGFR2 signalling [83]. In accordance with that, in the present study, lower plasma VEGF levels were found in rats fed with onion and apple supplemented diets, both diets rich in polyphenols, underlining their capability to modulate some factors involved in atherosclerosis. In this line, recently Roy et al. (2022) [84] reported that apple polyphenol phloretin complexed with ruthenium is capable of reprogramming the breast cancer microenvironment through modulation of PI3K/Akt/mTOR/VEGF pathways.

In obese animals, the dysregulated endocrine function of adipose tissue, particularly the visceral compartment, leads to an increased release of hormones and pro/antiinflammatory molecules. These molecules include adhesion molecules such as intercellular cell adhesion molecule-1 (ICAM-1) and E-selectin, chemokines (such as MCP-1) and adipokines (such as adiponectin). Adhesion molecules play a central role in adherence of cells to endothelial surfaces, in the integrity of the vascular wall and can be modulated by dietary pattern and body composition [85]. In the present study, sICAM-1, E-selectin and MCP-1 were significantly lower in obese rats fed the onion and apple diets compared with the obese rats in accordance with previous human and animal studies evaluating the role of different polyphenols and onion and apple formulations/diets/functional ingredients. Brüll et al. (2015) [86] reported a decrease in fasting serum sICAM-1 from baseline in overweight-to-obese patients with (pre-)hypertension supplemented with quercetin from onion skin extract, although no effect of quercetin supplementation on secondary variables such as endothelial function including reactive hyperaemia index, sE-selectin, sVCAM-1, asymmetric dimethylarginine and endothelin-1 were found. Mueller et al. (2013) [87] studied the influence of triterpenoids present in apple peel on inflammatory gene expression associated with inflammatory bowel disease; however, the influence of apple constituents on inflammatory gene expression is mainly attributed to several active phenolic constituents, like procyanidins and phloretin and phlorizin derivates. In this regard, phlorizin dietary treatment in high-fat diet fed mice for 6 weeks significantly reduced concentration of serum and adipose tissue proinflammation cytokines, including MCP-1, TNF-α, IL-1, IL-6 and IL-1β without altering body fat and serum lipid levels [88]. Phloretin prevented high-fat diet-induced obesity and improved metabolic homeostasis by suppressing the expression of macrophage markers (F4/80 and Cd68) and proinflammatory genes (Mcp-1 and Ccr2) and enhancing adiponectin gene expression in white adipose tissue. In addition, phloretin treatment elevated the expression of fatty acid oxidation genes such as carnitine palmitoyltransferase 1a and 1b (Cpt1a and Cpt1b) and reduced expression of monocyte chemoattractant protein-1 (Mcp-1) in obese mice fed a high-fat diet [89]. Based on these studies, an involvement in the decrease in inflammatory biomarkers by these compounds identified and quantified in the onion and apple lyophilized powder used for the preparation of the diets can be hypothesized. Adiponectin levels have been reported to be negatively correlated with cancer, cardiovascular disease and diabetes, and shown to be affected (i.e., significantly increased) by proper healthy nutrition. Accordingly, in the present study, plasma adiponectin concentration was significantly lower in obese rats compared to lean rats. However, no significant effect was found in rats fed the functional ingredients, as no differences were detected between the obese group and the groups fed the onion and apple powder. In line with the present study for adiponectin results, Brüll et al. (2017) [90] did not find effects of quercetin from onion skin extract on serum leptin and adiponectin concentrations in overweight-to-obese patients with (pre-)hypertension, suggesting that the supplementation period, which was only for 6 weeks, was possibly not long enough to observe changes in the markers of systemic and adipose tissue-associated inflammation, as could be one of the reasons also in the present study. Also, Kim et al. (2016) [91] suggested that 12-week supplementation of a quercetin-rich onion peel extract (OPE) does not affect modulators of systemic inflammation in overweight and obese women since ALT and AST activities, as well as leptin, adiponectin, visfatin, TNF-α, and IL-4 levels in plasma, were not significantly different between placebo and OPE supplementation groups compared with baseline value after 12 weeks of the supplementation. In this line, Eisner et al. (2020) [92] reported no significant differences in glucose, insulin, proinsulin, HOMA-IR, CRP, or adiponectin after dried apple consumption for 8 weeks in overweight and obese children. In addition, in a study evaluating the effects of the administration of different soluble fibre enriched-diets on inflammatory and redox state of Zucker fatty rats, adiponectin plasma levels increased in the Zucker fatty rats fed the soluble cocoa fibre-enriched diet, but did not change in the rats fed the high methoxylated apple pectin- and β-glucan-enriched diets [93]. In contrast, Sung et al. (2011) [94] reported that the concentration of serum adiponectin was significantly increased in the *Allium fistulosum* L extract-treated group compared to the high-fat diet group in C57BL/6J mice, resulting in ameliorated insulin resistance, reduced serum lipid concentrations and body weight loss. In addition, Park et al. (2020) [95] reported that after the administration of cabbage–apple juice or fermented cabbage–apple juice in Sprague Dawley rats for 8 weeks showed a significant increase in adiponectin levels in the fermented cabbage–apple juice supplemented group, suggesting that fermented cabbage–apple juice acted to reduce body fat content because a significant reduction in total fat content was also observed. Accordingly, another reason that may explain the lack of effect on adiponectin by the onion and apple ingredients in the present study could be related to the lack of differences found in body weight gain in the three obese groups, as data in the literature report that adiponectin has a vital role in physiological neuromodulation of food intake, energy expenditure and body weight gain [96].

Regarding metabolic hormone concentration, plasma GLP-1 was lower in OC group than in LC, OO and OA groups. Plasma GLP-1 concentration increased by 41% and 22%, respectively, in OO and OA groups compared with OC group. Plasma leptin concentration was lower in LC group than in OC group (*p* < 0.05, Table 5). Leptin concentration was lower in OO and OA groups than in OC group (decrease by 10% and 7%, respectively). GLP-1 level was significantly lower in obese rats compared to lean rats and a significant increase was found in rats fed the functional ingredients compared to obese rats. The physiological role of GLP-1 is directed to control plasma glucose concentration; in fact, a clear association was found with the plasma glucose levels which were reduced in obese rats fed the ingredients compared to obese rats. Correspondingly, an improved insulin sensitivity (lower HOMA-IR) was observed with the feeding of onion and apple powder. In addition, these increased levels of GLP-1 may reflect an increased sensitivity of intestinal L cells to nutrients in obese rats fed the onion and apple ingredients. In line with these findings, Phuwamongkolwiwat et al. (2014) [97] conducted in vivo and in situ experiments with rats to investigate the GLP-1 secretion in response to oral or ileal administration of α-glycosyl-isoquercitrin (Q3G) and fructooligosaccharides (FOS). They found that although FOS alone did not have any effects, Q3G + FOS enhanced and prolonged a high plasma GLP-1 level in both experiments. The authors suggest that Q3G + FOS possess the potential to manage or prevent diabetes mellitus by improving and prolonging the GLP-1 secretion via direct stimulation of the GLP-1 producing L-cell. It has been reported that nutrients and other intestinal hormones act as potent stimulants of GLP-1 secretion, and high fibre diets have potential beneficial effects on gut hormones. In the present study, from the results achieved with onion and apple lyophilized powder (both rich in dietary fibre), it could be also extrapolated that modifying eating habits, food components, and some other factors to regulate GLP-1 levels may promote better management and treatment of obesity and diabetes which may be due to the lack of GLP-1, although the mechanism and long-term effectiveness of factors affecting the regulation of GLP-1 are still not fully understood. GLP-1 is reported to regulate food consumption through an interaction with leptin [98]. In accordance with the plasma GLP-1 concentrations, plasma leptin concentration was significantly higher in obese rats compared to lean rats and a significant decrease was found in rats fed the functional ingredients compared to obese rats. Leptin is a hormone secreted from the white adipose tissue and it especially regulates appetite. Leptin levels are increased by feeding and decreased by fasting, and the action of leptin is mediated through leptin receptors in the hypothalamus. In line with the present study, Sung et al. (2011) [94] reported a decrease in serum leptin levels in the *Allium fistulosum* L extract-treated group compared to the high-fat diet group in C57BL/6J mice. Raasmaja et al. (2013) [99] studied the effects of *Citrus grandis* (L.) Osbeck fruit extract on the energy metabolism in obese Zucker rats fed a high-fat diet, reporting that although not significant, the level of leptin decreased somewhat at 12 weeks in the citrus extract treated groups in a dose dependent manner. The authors suggest that the changes could be related with the increased food intake of those groups. Liou et al. (2020) [100] found that the administration of phloretin significantly inhibited the serum levels of leptin in obese mice, suggesting that this compound ameliorates hepatic steatosis through regulation of lipogenesis and Sirt1/AMPK signaling.

The current results regarding plasma inflammatory biomarkers are in agreement with a previous study investigating the effects of processed onion in hypercholesterolemic male Wistar rats. Inflammation and cardiovascular risk biomarkers (such as MCP-1, s-ICAM, sE-selectin, IL-10, VEGF, PAI-1, vWF and TIMPs) related to endothelial dysfunction and coagulation system were positively modulated by the onion intake, suggesting an atheroprotective effect induced by the diet enrichment with onion [24]. This could have translational relevance since increased VEGF-A serum levels have been shown to correlate with arterial stiffness in subjects with excess body mass [101] and have been proposed to intensify endothelial dysfunction in obese women [102]. Metabolomics shows itself to be a valuable tool for evaluating the impact of complementary dietetic approaches. In this sense, González-Peña et al. (2017) [103] carried out a non-targeted, multiplatform metabolomics approach to produce broad metabolite coverage in the liver of these rats. The model highlighted several metabolites (such as hydroxybutyryl carnitine and palmitoyl carnitine) modified by a high-cholesterol enriched with onion diet, suggesting potential impairments in the energy-lipid metabolism, perturbations in the tricarboxylic acid cycle cycle and β-oxidation modulated by the onion supplementation in the core of hepatic dysfunction. In the same animal model of hypercholesterolemia, onion supplementation modulated the hepatic concentrations of prostaglandins, modified the concentration of some CYP450/LOX derived oxylipins and increased ω-3-derived oxylipins in the liver, which encouraged further investigation of the effect of onion on pro-resolution pathways [104]. Regarding metabolic fate and cardiometabolic effects of phenolic compounds from apple, recently Yuste et al. (2021) [26] reported anthocyanins or flavan-3-ols, together with dihydrochalcones, compose a phenolic phytocomplex that could act synergistically in the attenuation of cardiovascular outcomes, such as the reduction of aorta thickness, the improvement of renal function and the reduction of insulin levels. These results could support the effects found in oxidative stress, inflammatory and vascular injury biomarkers in the current study. In fact, in a study from our research group using a dynamic gastrointestinal digestion model, the same high pressure-processed apple ingredient presented a higher recovery index for hydroxycinnamic acids and higher bioaccessibility for hydroxycinnamic acids, dihydrochalcones and total phenolic compounds compared to untreated apple, highlighting the relevance of the evaluation of the impact of gastrointestinal digestion on the stability and bioaccessibility of phenolic compounds in order to further investigate their biological activity [105]. In addition, food matrix and processing parameters in the preparation of onion and apple lyophilized powder need to be taken into account in the evaluation of phenolic compounds’ bioaccessibility as responsible in part for their beneficial health effects [106].

### 3.4. Vascular Reactivity in Mesenteric Arteries

**Figure 1:**
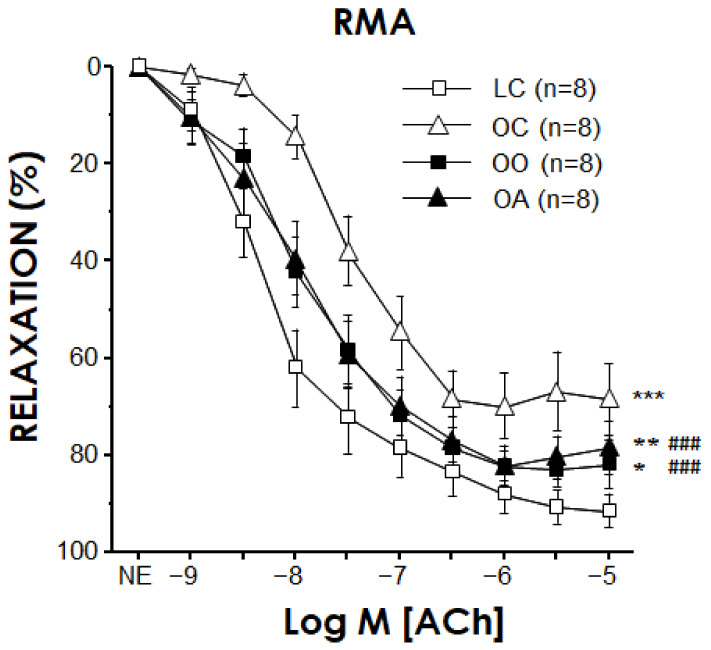
Onion and apple ingredients improve endothelium-dependent vasodilation in mesenteric arteries (RMA) from obese Zucker rats. Endothelium-dependent relaxation induced by acetylcholine (ACh) in RMA precontracted with norepinephrine (NE) obtained from lean Zucker rats fed a standard diet (lean control group: LC), obese Zucker rats fed a standard diet (obese control group: OC), obese Zucker rats fed a standard diet containing 10% onion (obese onion 10% group: OO) and obese Zucker rats fed a standard diet containing 10% apple (obese apple 10% group: OA). Data are expressed as mean ± SEM of the percentage of maximal relaxation induced by papaverine (0.1 mM) at the end of the experiment. *n* indicates the number of rats. * *p* < 0.05, ** *p* < 0.01, *** *p* < 0.001 vs. LC, ### *p* < 0.001 vs. OC by a two-factors ANOVA test and Bonferroni correction.

ACh (1 nM to 30 μM) caused endothelium-dependent vasodilation in RMA which was significantly impaired in obese Zucker rats fed a standard diet (pEC_50_ for ACh 7.91 ± 0.19 vs. 6.77 ± 0.39 for LC and OC, respectively, *p* < 0.01). This endothelial impairment was partially reversed in a similar magnitude by onion and apple ingredients (pEC_50_ for ACh 7.64 ± 0.24 vs. 7.68 ± 0.24 for OO and OA, respectively, both *p* < 0.05 vs. OC) (Figure 1). Precontractile tone induced by NE (determined as the percentage of KKHS-induced contraction) was not significantly modified by obesity nor the diets (82.4 ± 5.0%, 92.3 ± 5.7%, 88.0 ± 5.6% and 83.8 ± 7.7% for LC, OC, OO and OA, respectively).

On the other hand, endothelium-independent relaxation induced by SNP (1 nM to 100 μM) was not significantly altered in this model of obesity. In the same way, parameters of SNP-induced relaxations were not significantly modified by onion or apple enriched diets (pEC_50_ for SNP 6.67 ± 0.31, 6.72 ± 0.50, 5.98 ± 0.31 and 6.80 ± 0.39 for LC (*n* = 8), OC (*n* = 8), OO (*n* = 7) and OA (*n* = 7), respectively, n.s.; E_max_ for SNP 96.6 ± 1.0%, 91.7 ± 3.8%, 93.3 ± 2.9% and 96.6 ± 1.5% for LC, OC, OO and OA, respectively, n.s.).

**Figure 2:**
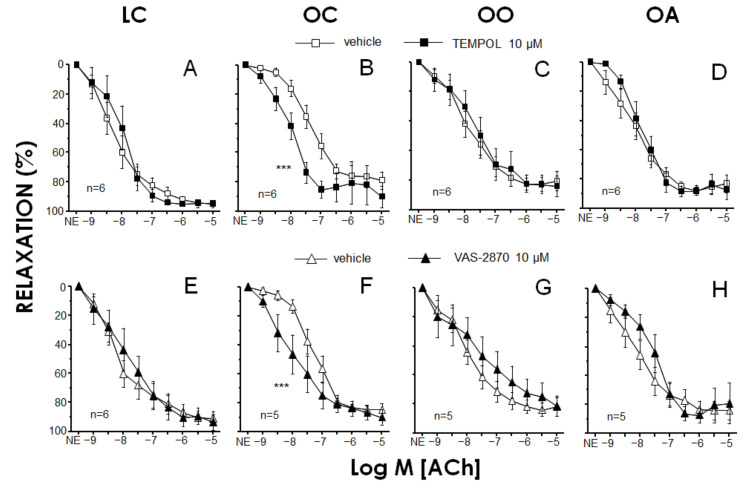
Increased superoxide generation from NADPH oxidase is responsible for endothelial dysfunction in mesenteric arteries (RMA) from obese Zucker rats but this effect is prevented by onion and apple ingredients. Effects of the superoxide dismutase analogue (TEMPOL, 10 μM) (**A**–**D**) and the NADPH oxidase inhibitor (VAS-2870, 10 μM) (**E**–**H**) on endothelium-dependent relaxation induced by acetylcholine (ACh) in RMA precontracted with norepinephrine (NE) obtained from lean Zucker rats fed a standard diet (lean control group: LC), obese Zucker rats fed a standard diet (obese control group: OC), obese Zucker rats fed a standard diet containing 10% onion (obese onion 10% group: OO) and obese Zucker rats fed a standard diet containing 10% apple (obese apple 10% group: OA). Data are expressed as mean ± SEM of the percentage of maximal relaxation induced by papaverine (0.1 mM) at the end of the experiment. *n* indicates the number of rats. *** *p* < 0.001 vs. vehicle by a two-factors ANOVA test.

The effects of superoxide scavenging and inhibition of NOX on endothelium-vasodilations in RMA from different animal groups were evaluated by preincubating the vessels with TEMPOL (10 μM) and VAS-2870 (10 μM), respectively (Figure 2). Neither TEMPOL (89.0 ± 10.6%, 87.0 ± 15.5%, 92.5 ± 3.2% and 89.8 ± 8.4% for LC, OC, OO and OA, respectively) nor VAS-2870 (79.5 ± 4.6%, 93.2 ± 9.8%, 87.2 ± 9.1% and 84.7 ± 12.0% for LC, OC, OO and OA, respectively) affected contractile tone induced by NE in RMA from any of the groups. In contrast, TEMPOL caused a significant increase in ACh-induced relaxations in RMA from OC rats (Figure 2B) but failed to exert any significant effect on endothelium-dependent relaxation in arteries from LC, OO or OA rats (Figure 2A,C,D). Analogously, the NOX inhibitor VAS-2870 did not significantly modify ACh-induced relaxation in RMA from LC, OO or OA, but enhanced endothelial vasodilation in mesenteric vessels from OC (Figure 2E–H). The specific potentiation of endothelial vasodilation by TEMPOL in OC rats is suggestive of an involvement of superoxide anion in endothelial dysfunction in these animals, while the similar effect driven by the NOX inhibitor VAS-2870 points to a main contribution of NOX enzymes to the superoxide generation leading to impaired endothelial vasodilation in obese rats. The participation of ROS in the impairment of endothelial relaxations in obese Zucker rats has been previously proposed in different vascular territories, including mesenteric arteries [107,108,109], as well as in obese subjects with insulin resistance [110]. Moreover, an involvement of NOX-1 in endothelial impairment has been reported in renal arteries from obese Zucker rats [111]. Lack of effect of the superoxide modulators in arteries from OO and OA rats would indicate that a potential increase in superoxide related to obesity was prevented by the chronic treatment with onion and apple extracts.

The endothelial impairment partially reversed by onion and apple powder intake could be attributed to the vascular-protective role of polyphenols (and especially their antioxidant properties) through the modification of the oxidative stress status [112,113]. For example, there is scientific evidence in experimental studies about the quercetin direct acute vasodilator effects in isolated arteries and prevention of endothelial dysfunction, superoxide production and overexpression of p47 phox induced by angiotensin II in rat aorta [114]. Another study highlighted a significant restoration of ACh-induced endothelium-dependent relaxation in the aorta isolated from catechin hydrate-treated diabetic rats. However, catechin hydrate-induced restoration of ACh-provoked endothelium-dependent relaxation in the aorta of diabetic rats was significantly attenuated upon the incubation of the aortic ring with either L-NAME or Wortmannin [115]. In line with the current findings, consumption of onion peel extract induced an improvement in endothelial function and circulating endothelial progenitor cells levels in healthy overweight and obese individuals [116], and chronic onion extract intake ameliorated postprandial endothelial dysfunction in healthy men [117]. In addition, Felice et al. (2019) [118] reported that 4 weeks’ consumption of extra virgin olive oil-enriched dark chocolate would improve endothelial function, expressed as an increase in the median levels of circulating endothelial progenitor cells number in patients with cardiovascular risk factors; however, apple-enriched dark chocolate consumption was not effective. On the contrary, a study in healthy male Kunming mice found that apple peel polyphenols exerted a protective effect against high-cholesterol-induced endothelial dysfunction and hepatotoxicity in mice [119], and a study in male C57Bl6J mice knockout for apolipoproteinE (ApoE^−^/^−^) showed that endothelial dysfunction was prevented when the animals were fed a Western diet with sunflower oil (n-6 PUFAs) supplemented with both apple puree and polyphenol extract (the maximal response to ACh was increased by 40%). An interesting point is that contrary to the Western diet plus n-6 PUFAs group which presented an impairment of vasodilation to ACh in 75% of the tested arteries, no impaired aorta was reported in both supplemented groups, and no impact on the dose-response to SNP was observed [120]. Vendrame et al. (2014) [121] found that a wild blueberry (source of anthocyanins and other polyphenols) diet partially restored phenylephrine-induced constrictor responses and attenuated acetylcholine-induced relaxant responses in obese Zucker rats. Previous results from our research group showed that the onion ingredient reduced the increment in NOX activity and reversed endothelial dysfunction promoted by the high-cholesterol diet in Wistar rats [39], underlining the cardioprotective effects of specific phytochemicals (flavonols and sulphur compounds) and nutritional compounds (dietary fibre) present in onion.

### 3.5. Detection of Superoxide Anion Generation

**Figure 3:**
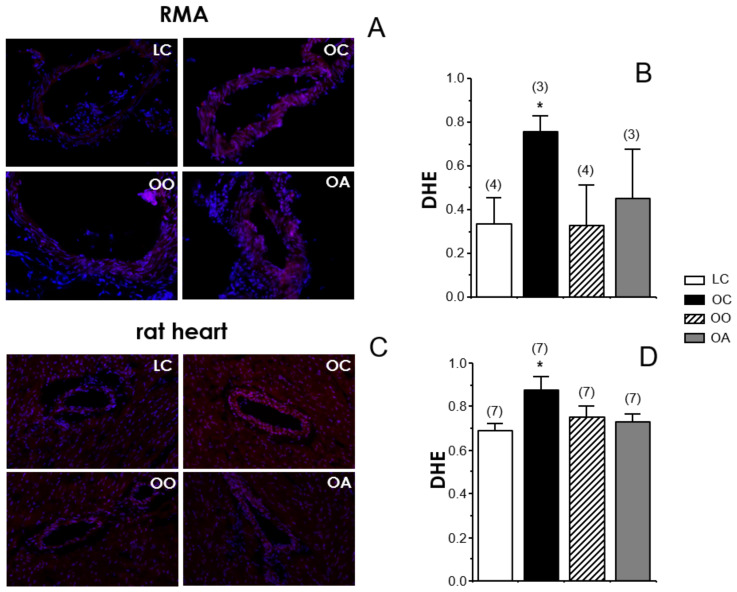
Onion and apple ingredients decrease superoxide anion content in mesenteric arteries (RMA) and hearts (including left coronary artery) from lean and obese Zucker rats. Representative images (×200) of immunodetection of superoxide content detected by dihydroethidium (DHE)-induced fluorescence (red) in mesenteric arteries (RMA) (**A**) and hearts (**C**) from lean Zucker rats fed a standard diet (lean control group: LC), obese Zucker rats fed a standard diet (obese control group: OC), obese Zucker rats fed a standard diet containing 10% onion (obese onion 10% group: OO) and obese Zucker rats fed a standard diet containing 10% apple (obese apple 10% group: OA). Nuclei staining with DAPI (blue) are merged in all images. Positive nuclei yielded purple colour. Right panels show fluorescence quantification in RMA (**B**) and heart (**D**). Data are expressed as mean ± SEM of the ratio of nuclei stained with DHE with respect to total number of nuclei. Number of rats is indicated in parentheses. * *p* < 0.05 vs. LC by one-factor ANOVA followed by Student–Newmann–Keuls test.

The oxidative stress status was evaluated by detecting superoxide content with DHE probe in RMA and hearts from all the experimental rat groups. The amount of superoxide anion detected by the fluorescent probe, DHE, was significantly increased in RMA from OC rats. However, this increase in superoxide content was not detected in RMA from OO and OA rats (Figure 3A). The results in RMA (Figure 3A,B) were qualitatively similar to those obtained in hearts (Figure 3C,D) from these animals where feeding with onion or apple ingredients prevented the increase of superoxide content observed in untreated obese Zucker rats. These data are consistent with the functional results of the endothelium-dependent vasodilation in RMA. In this sense, the normalization of superoxide content in cardiovascular tissues from obese Zucker rats by onion and apple supplementation could explain the improvement in endothelial vasodilation by these treatments (Figure 1) and the absence of further potentiation of endothelial relaxations by the acute exposure to the superoxide scavenger or the NOX inhibitor in vessels from supplemented animals (Figure 2). Elevated superoxide content has been detected in arteries from obese mice associated with decreased NO content [122]. Furthermore, in obese humans with insulin resistance, the increase in superoxide content in mesenteric microvasculature correlated with a reduction in endothelial vasodilation [110].

Onion and apple antioxidants and related bioactive compounds have influenced the ROS production; these findings are in line with the higher plasma ABTS^•^^+^ and FRAP, plasma nitrate/nitrite, erythrocyte SOD and GPx activities and GSH/GSSH ratio in rats fed the onion and apple diets compared to those of the obese control group. In accordance with the results of this study, we have previously shown a significant increase in the NOX activity in arteries obtained from a high-cholesterol-fed rats group, which was significantly reduced in vessels from rats fed the high-cholesterol enriched with onion diet. Also, recently Rodríguez-Rodríguez et al. (2022) [123] indicated the capacity of cocoa shell extract to reduce superoxide anion levels in aged rats. Additionally, Rozentsvit et al. (2017) [124] found the ellagic acid significantly decreased endothelial ROS levels and ameliorated the impairment of vascular relaxation induced by high glucose in Sprague–Dawley rats, suggesting that polyphenols exert a vasculo-protective effect under diabetic conditions via an antioxidant effect that involves inhibition of ERK1/2 and downregulation of NOX4. However, the fact that the improvement in endothelial vasodilation by onion and apple supplementations did not achieve a complete recovery would suggest the contribution of additional mechanism(s) to the defective endothelial function in obese Zucker rats which is(are) not influenced by these dietary approaches.

### 3.6. RNA Isolation and Quantitative Real Time PCR (RT-PCR) Assay

**Figure 4:**
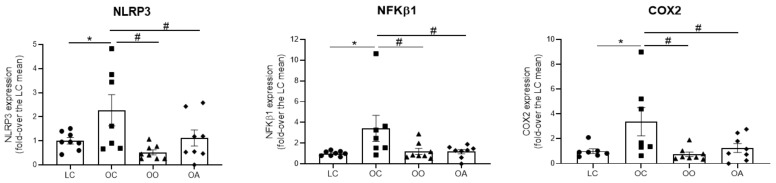
Onion and apple ingredients decrease gene expression of NLRP3, NFKβ1 and COX2 measured in aortic tissues using qRT-PCR from lean Zucker rats fed a standard diet (lean control group: LC), obese Zucker rats fed a standard diet (obese control group: OC), obese Zucker rats fed a standard diet containing 10% onion (obese onion 10% group: OO) and obese Zucker rats fed a standard diet containing 10% apple (obese apple 10% group: OA). Data are expressed as mean ± SEM of five independent experiments. * *p* < 0.05 vs. LC; # *p* < 0.05 vs. OC by one-way ANOVA followed by Sidak post-hoc test.

Gene expression of NLRP3, NFKβ1 and COX2, all of them players in inflammatory responses, were measured in aortic tissues using qRT-PCR. Nod-like receptor protein 3 (NLRP3) is the best characterized sensor protein of the inflammasome pathway, a first-line player of the innate immune response. The aberrant over-activation of the NLRP3 inflammasome has been linked with several inflammatory disorders, including vascular disease and atherosclerosis, or obesity-induced inflammation and insulin resistance [125]; nuclear factor-kappa B1 (NFKB1) is a ubiquitous transcription factor and a master driver of inflammatory and immune responses; and COX2 is an enzyme involved in inflammatory processes, and a main producer of prostanoids, including thromboxane A2 (TXA2), which is associated with inflammation and premature ageing. The results indicated that OC rats showed a significantly higher gene expression by 2.60 ± 0.64, 3.42 ± 1.25, and 3.37 ± 1.14 fold-increase when compared with LC for NLRP3, NFKβ1 and COX2, respectively. However, this effect was equally prevented in rats fed the onion ingredient (0.52 ± 0.10, 1.20 ± 0.29 and 0.74 ± 0.18 fold-increase, respectively, in OO rats) or rats fed the apple ingredient (1.11 ± 0.33, 1.17 ± 0.21 and 1.25 ± 0.35 fold-increase, respectively, in OA rats) (Figure 4).

The over-activation of NLRP3 inflammasome, NF-κB and COX2 in OC may have a common basis in the enhanced production of oxidative stress. Indeed, ROS positively regulate the activity of NLRP3 inflammasome and the subsequent release of proinflammatory cytokines such as IL-1β and IL-18 [126]. Moreover, NF-κB is a transcription factor sensitive to ROS and it induces proinflammatory enzymes, including COX-2 [127]. By decreasing oxidative stress, onion and apple supplementations may help preventing the proinflammatory activation observed in OC vessels. Interestingly, and in line with the present findings, Jiang et al. (2022) [79] recently reported that Siberian onion reduced the positive expressions of α-smooth muscle actin and NLRP3 by inhibiting signal transducer and activator of transcription 3 phosphorylation in activated hepatic stellate cells. Moreover, a study by Guo et al. (2013) [128] concluded that quercetin and quercetin-3-*O*-glucuronide inhibited ROS-associated inflammation by blocking IKKβ/NF-κB activation and effectively facilitated insulin signaling transduction along insulin receptor substrate-1/Akt/eNOS pathway in the endothelium. On the other hand, Xu et al. (2015) [129] confirmed that the apple polyphenols greatly reduced the ox-LDL-induced endothelial dysfunction and monocyte adhesion to rat aortic endothelial cells, suggesting that mechanistically, the apple polyphenols treatment suppressed the ROS/MAPK/NF-κB signaling pathway, and consequently, reduced CCL-2, ICAM-1 and VCAM-1 expression. Moreover, rats consuming Western diet/10% apple pomace had downregulated hepatic and adipose proinflammatory cytokine gene expression and improved antioxidant status compared to rats consuming a Western diet [17]. Xiao et al. (2014) [130] showed that epigallocatechin gallate (catechin present in apple) reduced the severity of liver injury in an experimental model of NAFLD associated with lower concentration of pro-fibrogenic, oxidative stress (e.g., nitrotyrosine formation) and proinflammatory mediators (e.g., iNOS, COX2, and TNF-α), partly through modulating the activities of TGF/SMAD, PI3 K/Akt/FoxO1 and NF-κB pathways. From the results presented and the scientific literature it could be extrapolated that apple polyphenols could help prevent cardiovascular disease and type 2 diabetes mellitus by mediating the AMPK pathway, the Nrf2 signaling pathway, the polyol pathway and the NF-κB pathway [23]. In the present study, following onion and apple consumption, COX2 expression in the aorta was significantly downregulated, reflecting the antioxidant and antiinflammatory effects of onion and apple bioactive compounds on NF-κB activation. In agreement with these results, in the study by Vendrame et al. (2014) [121], downregulation of inducible nitric oxide synthase and COX2 expression in the aorta of obese Zucker rats was observed in the rats fed the wild blueberry diet, pointing out the role of polyphenol-rich diets in inflammation and vascular function.

Strengths of the present study include the use of an advanced technology for obtaining the here exhaustively characterized onion and apple lyophilized powder, the use of a well-established rat model of obese metabolic syndrome and the extensive determination of food parameters as well as metabolic, antioxidant/oxidative stress and inflammatory biomarkers. Additionally, the study provides a demonstration of the beneficial effects of onion and apple supplementation on endothelial function and on oxidative stress and inflammation in vascular tissues from obese animals. The study has the obvious limitation of an animal study when translation to human subjects is the main aim. In addition, the present study cannot ascertain the exact biologically active substance(s) contained in onion and apple diets responsible for the described effects.

## 4. Conclusions

The results reported here show for the first time that the intake of lyophilized powder obtained from high pressure-processed onions and apples in obese Zucker rats causes beneficial effects on metabolic health and vascular health. It improves food efficiency, lipid profile and glucose metabolism, and reduces biomarkers of hepatic injury. Metabolic benefits are accompanied by systemic improvement of oxidative stress, inflammation and vascular injury biomarkers. At vascular level, onion and apple powder improves endothelial function and decreases oxidative stress and proinflammatory gene expression and reduces the activation of key components of the innate immune response. Therefore, onion and apple have a great potential for their use in functional ingredients able to induce antiobesity effects and beneficially modulate cardiovascular risk factors. In this sense, further research is needed to ascertain the polyphenols and other biologically active substances that reach circulation and target tissues and may be responsible for the health effects derived from their consumption.

## Figures and Tables

**Table 1:** Nutritional composition, phytochemical compounds and antioxidant activity of onion and apple powder.

	Onion Powder	Apple Powder
Protein (g/100 g)	9.87 ± 0.07	1.60 ± 0.01
Lipids (g/100 g)	1.35 ± 0.05	1.45 ± 0.07
Carbohydrates (g/100 g)	79.12 ± 3.18	85.67 ± 3.84
Glucose (g/100 g)	32.81 ± 1.25	15.23 ± 1.81
Fructose (g/100 g)	19.04 ± 2.50	31.37 ± 3.08
Sucrose (g/100 g)	7.74 ± 1.28	5.93 ± 0.85
Total dietary fibre (g/100 g)	16.86 ± 1.23	14.43 ± 1.89
Soluble fibre (g/100 g)	3.71 ± 0.07	3.14 ± 0.56
Insoluble fibre (g/100 g)	13.15 ± 0.97	11.29 ± 1.63
Pectin (g/100 g)	ND	2.67 ± 0.67
Ash (g/100 g)	3.59 ± 0.15	0.98 ± 0.04
Total phenols (mg GAE/100 g)	948.78 ± 39.75	2077.03 ± 74.42
Quercetin (mg/100 g)	1.56 ± 0.56	ND
Quercetin 3-O-rutinoside (mg/100 g)	ND	0.33 ± 0.07
Quercetin 3-O-arabinoside (mg/100 g)	ND	2.39 ± 0.30
Quercetin 3-O-xyloside (mg/100 g)	ND	3.82 ± 0.004
Quercetin 3-O-rhamnoside (mg/100 g)	ND	5.87 ± 0.05
Quercetin 3-O-glucoside (mg/100 g)	10.62 ± 0.08	2.45 ± 0.60
Quercetin 3-O-galactoside (mg/100 g)	ND	5.59 ± 0.79
Quercetin 4′-O-glucoside (mg/100 g)	313.53 ± 2.12	ND
Quercetin 3,4′-di-O-glucoside (mg/100 g)	1571.16 ± 6.75	7.44 ± 0.36
Quercetin 7,4′-O-diglucoside (mg/100 g)	37.95 ± 0.75	ND
Quercetin 3,7,4′-O-triglucoside (mg/100 g)	12.29 ± 0.35	ND
Isorhamnetin 3-O-glucoside (mg/100 g)	0.45 ± 0.001	ND
Isorhamnetin 4′-glucoside (mg/100 g)	12.95 ± 1.16	ND
Isorhamnetin 3,4′-diglucoside (mg/100 g)	3.18 ± 1.07	ND
Phloridzin (mg/100 g)	18.15 ± 1.57	6.35 ± 0.45
Phloretin (mg/100 g)	ND	0.17 ± 0.01
Phloretin 2′-xyloglucoside (mg/100 g)	ND	3.60 ± 0.14
Epicatechin (mg/100 g)	10.37 ± 0.35	20.81 ± 0.44
Catechin (mg/100 g)	ND	0.41 ± 0.004
Epicatechin dimer 1 (mg/100 g)	ND	4.05 ± 0.04
Epicatechin dimer 2 (mg/100 g)	ND	27.00 ± 0.90
Epigallocatechin (mg/100 g)	ND	11.18 ± 1.29
Procyanidin B2 (mg/100 g)	ND	26.84 ± 7.87
Chlorogenic acid (mg/100 g)	ND	28.15 ± 0.89
Neochlorogenic acid (mg/100 g)	ND	1.36 ± 0.003
Cryptochlorogenic acid (mg/100 g)	ND	1.67 ± 0.13
p-Coumaric acid (mg/100 g)	ND	0.08 ± 0.003
Propionaldehyde (mg/100 g)	15.59 ± 0.89	ND
1-Propanethiol (mg/100 g)	2.50 ± 0.15	ND
Hexanal (mg/100 g)	0.26 ± 0.02	2.17 ± 0.05
2-Methyl 2-pentenal (mg/100 g)	0.47 ± 0.05	ND
Propyl thioacetate (mg/100 g)	0.038 ± 0.0002	ND
Dimethyl trisulphide (mg/100 g)	0.057 ± 0.0004	ND
Dipropyl disulphide (mg/100 g)	2.70 ± 0.37	ND
Methyl propyl trisulphide (mg/100 g)	0.30 ± 0.02	ND
Dipropyl trisulphide (mg/100 g)	1.00 ± 0.09	ND
Ascorbic acid (mg/100 g)	58.75 ± 0.30	51.33 ± 0.80
Total vitamin C (mg/100 g)	71.25 ± 0.20	82.67 ± 0.90
Peroxidase activity (ΔOD/min/100 g)	7.66 ± 1.24	20.74 ± 0.89
Polyphenoloxidase activity (ΔOD/min/100 g)	0.38 ± 0.09	0.91 ± 0.04
ABTS^•^^+^ (μmol TE/100 g)	1509.45 ± 35.05	1821.79 ± 154.65
DPPH^•^ (μmol TE/100 g)	1129.50 ± 153.22	2589.74 ± 185.31
FRAP (μmol TE/100 g)	1122.55 ± 52.03	1582.95 ± 110.57

Data are presented as mean ± SD (*n* = 3). ND, not determined; GAE, gallic acid equivalents; ABTS^•+^, 2,2′-azinobis(3-ethylbenzothiazoline-6-sulfonic acid) radical cation; DPPH^•^, 2,2-diphenyl-1-picrylhydrazyl radical; FRAP, ferric reducing antioxidant power; TE, trolox equivalents.

**Table 2:** Composition of the experimental diets ^c^.

Component (g/kg)	LC/OC Diet	OO Diet	OA Diet
Onion powder	−	100	−
Apple powder	−	−	100
Casein	200	200	200
Sucrose	100	100	100
Maize starch	470.49	386.14	386.14
Soya oil	50	50	50
Maize oil	80	80	80
Mineral mixture ^a^	35	35	35
Vitamin mixture ^b^	10	10	10
Cellulose powder	50	34.35	34.35
Choline bitartrate	2.5	2.5	2.5
*tert*-butylhydroquinone	0.01	0.01	0.01
L-cystine	2	2	2

LC/OC, Lean control/Obese control diet; OO, Obese onion 10% diet; OA, Obese apple 10% diet. ^a^Mineral mix for the AIN-93M diet, g/kg (AIN-93M-MX): calcium carbonate anhydrous, 357.00; potassium phosphate monobasic, 250.00; potassium citrate, tripotassium monohydrate, 28.00; sodium chloride, 74.00; potassium sulphate, 46.00; magnesium oxide, 24.00; ferric citrate, 6.06; zinc carbonate, 1.65; sodium meta-silicate 9H_2_O, 1.45; manganous carbonate, 0.63; cupric carbonate, 0.30; chromium potassium sulphate 12H_2_O, 0.275; boric acid, 0.0815; sodium fluoride, 0.0635; nickel carbonate, 0.0318; lithium chloride, 0.0174; sodium selenate anhydrous, 0.01025; potassium iodate, 0.0100; ammonium paramolybdate 4H_2_O, 0.00795; ammonium vanadate, 0.0066; powdered sucrose, 209.806. ^b^Vitamin mix for the AIN-93M diet, g/kg (AIN-93M-VX): nicotinic acid, 3.000; calcium pantothenate, 1.600; pyridoxine-HCl, 0.700; thiamin-HCl, 0.600; riboflavin, 0.600; folic acid, 0.200; biotin, 0.200; vitamin B12 (cyanocobalamin) (0.1 % in mannitol), 2.500; vitamin E (all-*rac*-α-tocopheryl acetate, 500 IU/g), 15.000; vitamin A (all-*trans*-retinyl palmitate, 500,000 IU/g), 0.800; vitamin D3 (400,000 IU/g), 0.250; vitamin K1, 0.075; powdered sucrose, 974.655. ^c^Diet energy content was calculated using the factors 16.73 kJ/g (4 kcal/g) for protein, 15.69 kJ/g (3.75 kcal/g) for monosaccharides, 16.53 kJ/g (3.95 kcal/g) for disaccharides, 17.49 kJ/g (4.18 kcal/g) for starch, 8.37 kJ/g (2 kcal/g) for dietary fibre, and 37.65 kJ/g (9 kcal/g) for fat. LC/OC diet, 18,540.9 kJ/kg (4431.4 kcal/kg); OO diet, 18,424.8 kJ/kg (4403.6 kcal/kg); OA diet, 18,444.3 kJ/kg (4408.3 kcal/kg).

**Table 3:** Food intake, initial body weight, final body weight, body weight gain, food efficiency ratio, faecal weight and apparent diet digestibility in rats fed the lean control/obese control, obese onion 10% and obese apple 10% diets for 8 weeks.

	LC	OC	OO	OA
Food intake (g/day/rat)	18.44 ± 0.35 *^a^*	23.99 ± 1.23 *^b^*	24.98 ± 2.21 *^b^*	31.99 ± 1.07 *^c^*
Initial body weight (g)	197.75 ± 17.69 ^a^	221.88 ± 16.49 ^b^	225.63 ± 20.59 ^b^	226.88 ± 19.89 ^b^
Final body weight (g)	357.78 ± 38.45 ^a^	457.20 ± 20.10 ^b^	424.40 ± 30.03 ^b^	462.30 ± 51.55 ^b^
Body weight gain (g)	160.03 ± 27.08 ^a^	235.33 ± 15.80 ^b^	198.78 ± 27.68 ^b^	235.43 ± 40.12 ^b^
Food efficiency ratio ^†^	15.49 ± 2.62 ^ab^	17.51 ± 1.17 ^b^	14.21 ± 1.98 ^a^	13.14 ± 2.24 ^a^
Faecal weight (g fresh matter/day)	2.91 ± 0.39 ^a^	3.89 ± 0.56 ^b^	3.77 ± 0.27 ^b^	4.11 ± 0.52 ^b^
Apparent diet digestibility ^§^	88.01 ± 3.50 ^a^	89.59 ± 1.72 ^a^	87.34 ± 1.71 ^a^	87.43 ± 1.97 ^a^

Data are presented as mean ± SD (*n* = 8/group). LC, Lean control group: lean Zucker rats fed a standard diet; OC, Obese control group: obese Zucker rats fed a standard diet; OO, Obese onion 10% group: obese Zucker rats fed a standard diet containing 10% onion; OA, Obese apple 10% group: obese Zucker rats fed a standard diet containing 10% apple. Mean values within a row with unlike superscript small letters were significantly different, *p* < 0.05 (one-way ANOVA and posterior Tamhane’s T2 and Bonferroni post hoc tests were used as appropriate, italic superscript small letters indicate Tamhane’s T2 post hoc test). ^†^ 100 × (body weight gain/total food intake). ^§^ 100 × (food intake–faecal weight/food intake).

**Table 4:** Plasma lipid concentrations and biochemical parameters in rats fed the lean control/obese control, obese onion 10% and obese apple 10% diets for 8 weeks.

	LC	OC	OO	OA
Total cholesterol (mmol/l)	2.65 ± 0.33 *^a^*	6.93 ± 1.80 *^c^*	4.53 ± 1.18 *^b^*	5.18 ± 1.78 *^bc^*
HDL-cholesterol (mmol/l)	1.50 ± 0.22 ^a^	2.66 ± 0.76 ^b^	3.52 ± 0.78 ^c^	2.84 ± 0.39 ^bc^
LDL-cholesterol (mmol/l)	0.28 ± 0.003 ^a^	1.03 ± 0.11 ^c^	0.89 ± 0.08 ^b^	0.98 ± 0.09 ^bc^
AI (1)	0.19 ± 0.02 *^a^*	0.41 ± 0.11 *^c^*	0.26 ± 0.04 *^b^*	0.35 ± 0.04 *^c^*
AI (2)	1.78 ± 0.21 *^b^*	2.66 ± 0.45 *^c^*	1.29 ± 0.18 *^a^*	1.80 ± 0.49 *^b^*
Triacylglycerols (mmol/l)	3.81 ± 0.82 *^a^*	35.77 ± 3.89 *^c^*	28.05 ± 4.42 *^b^*	32.55 ± 2.82 *^bc^*
Glucose (mmol/l)	7.16 ± 1.61 *^a^*	26.20 ± 2.94 *^c^*	17.14 ± 6.74 *^b^*	20.19 ± 7.63 *^bc^*
Insulin (pmol/l)	88.31 ± 12.50 *^a^*	651.04 ± 220.96 *^c^*	332.26 ± 124.17 *^b^*	430.25 ± 150.88 *^bc^*
HOMA-IR	3.95 ± 0.42 *^a^*	112.26 ± 48.25 *^d^*	31.91 ± 7.22 *^b^*	50.30 ± 14.82 *^bc^*
Triglyceride–Glucose Index	9.94 ± 0.34 *^a^*	13.51 ± 0.17 *^c^*	12.76 ± 0.54 *^b^*	13.08 ± 0.47 *^bc^*
Glucose/Insulin Ratio	10.57 ± 3.84 ^a^	5.54 ± 1.80 ^a^	8.67 ± 6.99 ^a^	7.40 ± 5.46 *^a^*
Urea (mmol/l)	13.39 ± 0.89 *^a^*	12.99 ± 2.96 *^a^*	12.09 ± 3.03 *^a^*	11.20 ± 3.15 *^a^*
Uric acid (µmol/l)	31.23 ± 10.42 ^a^	32.71 ± 11.90 ^a^	23.79 ± 6.36 ^a^	23.05 ± 7.41 ^a^
Creatinine (µmol/l)	59.67 ± 4.09 ^a^	78.45 ± 22.88 ^a^	75.14 ± 29.51 ^a^	81.77 ± 34.96 ^a^
Albumin (g/l)	40.75 ± 1.98 *^a^*	55.87 ± 9.52 *^b^*	55.50 ± 9.69 *^b^*	57.62 ± 9.88 *^b^*
ALT (U/l)	77.25 ± 19.56 *^a^*	170.12 ± 43.09 *^b^*	90.12 ± 26.35 *^a^*	104.25 ± 23.19 *^a^*
AST (U/l)	284.50 ± 55.46 ^a^	459.12 ±72.97 ^b^	333.87 ± 40.48 ^a^	344.37 ± 48.59 ^a^
GGT (U/l)	13.60 ± 1.94 ^a^	29.42 ± 3.62 ^c^	22.85 ± 1.78 ^b^	25.01 ± 1.73 ^b^
Total bilirubin (µmol/l)	18.38 ± 5.31 ^a^	33.77 ± 9.08 ^b^	31.85 ± 5.92 ^b^	26.50 ± 5.71 ^b^

Data are presented as mean ± SD (*n* = 8/group). LC, Lean control group: lean Zucker rats fed a standard diet; OC, Obese control group: obese Zucker rats fed a standard diet; OO, Obese onion 10% group: obese Zucker rats fed a standard diet containing 10% onion; OA, Obese apple 10% group: obese Zucker rats fed a standard diet containing 10% apple. Mean values within a row with unlike superscript small letters were significantly different, *p* < 0.05 (one-way ANOVA and posterior Tamhane’s T2 and Bonferroni post hoc tests were used as appropriate, italic small letters indicate Tamhane’s T2 post hoc test). AI (1), Atherogenic index (1): LDL-cholesterol/HDL-cholesterol; AI (2): Total cholesterol/HDL-cholesterol; HOMA-IR: [Fasting Insulin (microU/mL) × Fasting Glucose (mmol/L)]/22.5; Triglyceride–Glucose Index: Ln[Fasting Plasma Triacylglycerols (mg/dL) × Fasting Glucose (mg/dL)/2]; Glucose/Insulin Ratio: Fasting Glucose (mg/dL)/Fasting Insulin (microU/mL).

**Table 5:** Antioxidant, oxidative stress, inflammatory and vascular injury biomarkers and metabolic hormones in rats fed the lean control/obese control, obese onion 10% and obese apple 10% diets for 8 weeks.

	LC	OC	OO	OA
Plasma ABTS^•+^ (μmol TE/L)	1727.84 ± 85.39 ^b^	1504.42 ± 44.88 ^a^	1686.67 ± 24.73 ^b^	1757.08 ± 77.48 ^b^
Plasma FRAP (μmol TE/L)	828.61 ± 59.87 ^b^	581.59 ± 54.25 ^a^	872.99 ± 57.30 ^b^	843.04 ± 55.98 ^b^
Erythrocyte catalase (CAT) activity (nmol/min/mL)	1759.24 ± 12.06 ^b^	1696.97 ± 28.65 ^a^	1788.10 ± 5.18 ^c^	1696.27 ± 8.69 ^a^
Liver catalase (CAT) activity (nmol/min/mg protein)	390.81 ± 36.24 ^b^	188.15 ± 6.39 ^a^	206.02 ± 24.17 ^a^	194.01 ± 8.20 ^a^
Erythrocyte superoxide dismutase (SOD) activity (U/mL)	117.83 ± 2.87 ^b^	104.10 ± 2.68 ^a^	143.07 ± 0.61 ^c^	123.32 ± 14.50 ^b^
Liver superoxide dismutase (SOD) activity (U/mg protein)	0.014 ± 0.002 *^c^*	0.0042 ± 0.0005 *^ab^*	0.0046 ± 0.004 *^b^*	0.0037 ± 0.001 *^a^*
Erythrocyte glutathione peroxidase (GPx) activity (nmol/min/mL)	35.66 ± 3.85 ^c^	17.93 ± 0.17 ^a^	25.67 ± 1.02 ^b^	22.92 ± 2.26 ^b^
Liver glutathione peroxidase (GPx) activity (nmol/min/mg protein)	2.42 ± 0.08 ^c^	1.61 ± 0.10 ^a^	2.12 ± 0.11 ^b^	2.06 ± 0.02 ^b^
Erythrocyte glutathione/glutathione disulphide (GSH/GSSH) ratio	0.49 ± 0.01 ^c^	0.31 ± 0.03 ^a^	0.45 ± 0.02 ^b^	0.52 ± 0.02 ^d^
Liver glutathione/glutathione disulphide (GSH/GSSH) ratio	3.59 ± 0.16 ^c^	1.75 ± 0.10 ^a^	2.25 ± 0.12 ^b^	2.21 ± 0.14 ^b^
Plasma protein carbonyls (nmol/mL)	21.52 ± 1.46 ^a^	29.89 ± 2.40 ^c^	25.45 ± 0.84 ^b^	21.36 ± 1.63 ^a^
Liver protein carbonyls (nmol/mL/mg protein)	0.97 ± 0.10 ^a^	1.71 ± 0.27 ^c^	1.28 ± 0.09 ^b^	1.30 ± 0.05 ^b^
Urine 8-hydroxy-2′-deoxyguanosine (ng/mL)-week 0	48.33 ± 0.75 ^aB^	54.19 ± 2.56 ^b*A*^	52.32 ± 0.82 ^bB^	52.97 ± 3.53 ^bB^
Urine 8-hydroxy-2′-deoxyguanosine (ng/mL)-week 8	45.02 ± 0.32 ^aA^	60.48 ± 0.64 ^c*B*^	46.29 ± 1.89 ^aA^	49.53 ± 1.08 ^bA^
Urine 8-*epi*-prostaglandin F_2α_ (pg/mL)-week 0	6.02 ± 1.05 ^aA^	7.79 ± 0.16 ^bA^	7.49 ± 0.41 ^bA^	7.39 ± 0.64 ^bA^
Urine 8-*epi*-prostaglandin F_2α_ (pg/mL)-week 8	12.03 ± 0.59 ^aB^	27.28 ± 0.32 ^bB^	11.65 ± 0.42 ^aB^	11.42 ± 0.73 ^aB^
Plasma nitrate/nitrite (µmol/mL)	55.70 ± 3.20 ^b^	36.35 ± 2.66 ^a^	55.53 ± 1.90 ^b^	55.22 ± 1.67 ^b^
Urine nitrate (NO_3_^−^)/nitrite (NO_2_^−^) (µmol/mL)-week 0	1072.78 ± 21.97 ^bB^	557.50 ± 37.86 ^aA^	574.17 ± 33.41 ^aA^	585.10 ± 7.36 ^aA^
Urine nitrate (NO_3_^−^)/nitrite (NO_2_^−^) (µmol/mL)-week 8	1024.17 ± 37.86 ^cA^	789.44 ± 36.00 ^aB^	1057.50 ± 20.04 ^cB^	907.50 ± 2.23 ^bB^
PAI-1 (ng/mL)	0.41 ± 0.02 ^a^	0.54 ± 0.03 ^c^	0.45 ± 0.03 ^ab^	0.46 ± 0.03 ^b^
TIMP-1 (ng/mL)	11.05 ± 0.76 ^a^	15.55 ± 1.38 ^c^	11.46 ± 1.08 ^ab^	13.03 ± 1.23 ^b^
VEGF (ng/mL)	0.13 ± 0.01 ^ab^	0.16 ± 0.01 ^c^	0.12 ± 0.006 ^a^	0.13 ± 0.004 ^b^
sICAM-1 (ng/mL)	0.0242 ± 0.001 ^a^	0.0419 ± 0.002 ^c^	0.0345 ± 0.002 ^b^	0.0245 ± 0.002 ^a^
sE-Selectin (ng/mL)	1.15 ± 0.09 ^a^	1.30 ± 0.05 ^b^	1.14 ± 0.09 ^a^	1.06 ± 0.10 ^a^
MCP-1 (pg/mL)	517.90 ± 30.22 ^ab^	761.27 ± 75.24 ^c^	570.24 ± 42.80 ^b^	506.16 ± 35.58 ^ab^
Adiponectin (ng/mL)	15.57 ± 1.05 ^b^	13.13 ± 0.41 ^a^	13.83 ± 0.79 ^a^	13.41 ± 0.92 ^a^
GLP-1 (pg/mL)	80.42 ± 3.65 ^d^	39.08 ± 2.53 ^a^	55.20 ± 2.52 ^c^	47.66 ± 3.60 ^b^
Leptin (pg/mL)	2516.58 ± 232.93 ^a^	7561.51 ± 529.90 ^c^	6813.08 ± 351.68 ^b^	7042.35 ± 274.07 ^b^

Data are presented as mean ± SD (*n* = 8/group). LC, Lean control group: lean Zucker rats fed a standard diet; OC, Obese control group: obese Zucker rats fed a standard diet; OO, Obese onion 10% group: obese Zucker rats fed a standard diet containing 10% onion; OA, Obese apple 10% group: obese Zucker rats fed a standard diet containing 10% apple. Mean values within a row with unlike superscript small letters were significantly different, *p* < 0.05 (one-way ANOVA and posterior Tamhane’s T2 and Bonferroni post hoc tests were used as appropriate, italic superscript small letters indicate Tamhane’s T2 post hoc test). Mean values within a column in the same group (LC, OC, OO, OA) for the same parameter with unlike superscript capital letters were significantly different, *p* < 0.05 (Student’s *t* test). Italic superscript capital letters indicate that equal variances were not assumed.

## Data Availability

Data are contained within the article.

## Abbreviations

ABTS^•+^: 2,2′-azinobis(3-ethylbenzothiazoline-6-sulfonic acid) radical cation; ACh: Acetylcholine; ALT: Alanine aminotransferase; AST: Aspartate aminotransferase; CAT: Catalase; COX2: Cyclooxygenase 2; DAPI: Diamidino-2-phenylindole; DPPH^•^: 2,2-diphenyl-1-picrylhydrazyl radical; EDTA: Ethylenediaminetetraacetic acid; FRAP: Ferric reducing antioxidant power; GAE: Gallic acid equivalents; GGT: Gamma-glutamyl transpeptidase; GLP-1: Glucagon-like peptide-1; GPx: Glutathione peroxidase; GSH/GSSH: Glutathione/glutathione disulphide ratio; HDL-C: HDL-cholesterol; HOMA-IR: Homeostatic model assessment of insulin resistance; KHS: Krebs-Henseleit solution; LC: Lean control; LDL-C: LDL-cholesterol; MCP-1: Monocyte chemoattractant protein-1; ND: Not determined; NE: Norepinephrine; NFKB1: Nuclear factor-kappa B1; NLRP3: Nod-like receptor protein 3; OA: Obese apple; OC: Obese control; OCT: Optimal cutting temperature; OO: Obese onion; PAI-1: Plasminogen activator inhibitor 1; RMA: Rat mesenteric arteries; SD: Standard deviation; SEM: Standard error of the mean; sICAM-1: Soluble intercellular adhesion molecule-1; SNP: Sodium nitroprusside; SOD: Superoxide dismutase; TC: Total cholesterol; TE: Trolox equivalents; TG: Triacylglycerols; TIMP-1: Tissue inhibitor of matrix metalloproteinases type 1; VEGF: Vascular endothelial growth factor.
